# The Prevalence of Metabolic Syndrome in the Different Phenotypes of Polycystic Ovarian Syndrome

**DOI:** 10.1055/s-0038-1676568

**Published:** 2019-01

**Authors:** Aleide Tavares, Romualda Castro Rêgo Barros

**Affiliations:** 1 Universidade Federal de Pernambuco, Recife, PE, Brazil

**Keywords:** polycystic ovarian syndrome, phenotype, insulin resistance, metabolic syndrome, abdominal obesity, síndrome do ovário policístico, fenótipo, resistência a insulina, síndrome metabólica, obesidade abdominal

## Abstract

**Objective** To evaluate the prevalence of metabolic syndrome (MetS) in the phenotypes of polycystic ovarian syndrome (PCOS).

**Methods** This was a cross-sectional study involving 111 women aged between 18 and 39 years old diagnosed with PCOS, according to the Rotterdam Criteria, and grouped into four phenotypes: A: ovulatory dysfunction + hyperandrogenism + polycystic ovaries; B: ovulatory dysfunction + hyperandrogenism; C: hyperandrogenism + polycystic ovaries; D: ovulatory dysfunction + polycystic ovaries. To evaluate the presence of MetS, we measured serum triglyceride levels, HDL cholesterol, fasting blood glucose, blood pressure, and waist circumference.

**Results** The prevalence of MetS found in this sample was 33.6%, and there was no statistically significant difference (*p* < 0.05) among the 4 phenotypes. However, phenotype D presented a significantly higher mean glucose level after fasting (93.6 mg/dL) and 2 hours after ingesting a solution with 75 g of anhydrous glucose (120 mg/dL), as well as the lowest mean level of high-density lipoprotein (HDL) cholesterol (44.7 mg/dL). The women in this group demonstrated a high prevalence of abdominal circumference ≥ 80 cm (68.2%), as well as the highest mean abdominal circumference (90.1 cm). Amongst the women with an abdominal circumference ≥ 80 cm, phenotype A increased approximately six-fold the chance of developing metabolic syndrome in relation to phenotype C.

**Conclusion** The four phenotypes of PCOS demonstrated similar prevalence rates of metabolic syndrome; abdominal obesity presented a relevant role in the development of metabolic alterations, regardless of the phenotype.

## Abstract (pt)

**Resumo:**

**Objetivo** Avaliar a prevalência da síndrome metabólica nos fenótipos da síndrome do ovário policístico.

**Métodos** Trata-se de um estudo transversal envolvendo 111 mulheres com idade entre 18 e 39 anos com diagnóstico de síndrome do ovário policístico, segundo os critérios de Roterdã, e agrupadas em quatro fenótipos: A: Disfunção ovulatória + hiperandrogenismo + ovários policísticos; B: disfunção ovulatória + hiperandrogenismo; C: hiperandrogenismo + ovários policísticos; D: disfunção ovulatória + ovários policísticos. Para avaliar a presença de síndrome metabólica, foram medidos os níveis séricos de triglicérides, colesterol HDL e glicemia de jejum, pressão arterial e circunferência da cintura.

**Resultados** A prevalência de síndrome metabólica encontrada nesta amostra foi de 33,6%, e não houve diferença estatisticamente significativa (*p* < 0,05) entre os quatro fenótipos. Entretanto, o fenótipo D apresentou um nível médio de glicose significativamente mais alto após o jejum (93,6 mg/dL) e duas horas após a ingestão de uma solução com 75g de glicose anidra (120 mg/dL), bem como o menor nível médio de colesterol HDL (44,7 mg/dl). As mulheres deste grupo demonstraram alta prevalência de circunferência abdominal ≥ 80 cm (68,2%), bem como a maior média de circunferência abdominal (90,1 cm). Entre as mulheres com circunferência abdominal ≥ 80 cm, o fenótipo A aumentou em aproximadamente 6 vezes a chance de desenvolver síndrome metabólica em relação ao fenótipo C.

**Conclusão** Os quatro fenótipos da síndrome do ovário policístico demonstraram taxas semelhantes de prevalência de síndrome metabólica; a obesidade abdominal apresentou papel relevante no desenvolvimento de alterações metabólicas, independentemente do fenótipo.

## Introduction

Polycystic ovarian syndrome (PCOS) is an endocrinopathy with manifestations of heterogeneous clinical signs and symptoms, such as hyperandrogenic disorders, oligomenorrhea or amenorrhea, infertility and obesity.^1^ ^2^ Polycystic ovarian syndrome is a complex syndrome, which presents different phenotypes.^2^ According to the Rotterdam diagnostic criteria, it is possible to identify the composition of four PCOS phenotypes: A: oligo-ovulation or anovulation + clinical and/or biochemical hyperandrogenism + polycystic ovaries; B: oligo-ovulation or anovulation + clinical and/or biochemical hyperandrogenism; C: clinical and/or biochemical hyperandrogenism + polycystic ovaries; D: oligo-ovulation or anovulation + polycystic ovaries. Environmental, cultural and genetic factors, as well as the diagnostic criteria used, also affect the prevalence rates of PCOS and its phenotypes. The literature states that the prevalence rates of PCOS vary between 2 and 20% in women of reproductive age.^3^ ^4^ ^5^ ^6^ ^7^ ^8^

Many studies have demonstrated that women with PCOS generally have a greater risk of developing cardiovascular disease and metabolic disorders when compared to control groups.^9^ ^10^ ^11^ ^12^ ^13^ ^14^ ^15^ The metabolic disorders of PCOS are mainly related to hyperandrogenism and compensatory hyperinsulinemia, and occur independently of obesity.^16^ ^17^ ^18^ However, little information is available as to whether cardiovascular risks are related with all the phenotypes of PCOS, the spectrum of which is broad and extends from women with evident signs of hyperandrogenism and amenorrhea to those who do not present with hyperandrogenism or present with regular cycles. It has recently been argued that, in terms of cardiovascular diseases and metabolic risks, not all women with PCOS should be considered equal.^19^ Metabolic disorders seem to be more prevalent in phenotypes A and B, that is, those considered as classic, followed by C (ovulatory), and much less frequently, D (nonhyperandogenic).^4^ ^20^ ^21^ ^22^ ^23^

The metabolic disorders presented in women with PCOS may make up metabolic syndrome (MetS), which is defined as the coexistence of risk factors for cardiovascular diseases in the same individual, with impaired glucose tolerance, dyslipidemia, and hypertension being the most relevant factors. Obesity, which is present in 30 to 70% of the cases of PCOS, presents an additive effect on metabolic risk factors, due to an exacerbation of insulin resistance (IR).^18^ Insulin resistance is considered to be a causal link between these factors and obesity, and is considered responsible for amplifying the reduction of tissue sensitivity to insulin.^13^ ^24^

It is essential to study the frequency of PCOS phenotypes, as well as their association with MetS in a given population group in order to help produce measures for the prevention and early treatment of cardiovascular diseases and type II diabetes.

## Methods

This was a descriptive, observational, cross-sectional study, conducted between June 2015 and November 2016, in the city of Recife, state of Pernambuco, Brazil. During this period, 163 women were referred from the primary health care services to the outpatient clinics of the Hospital Geral of the Universidade Federal de Pernambuco (HC-UFPE, in the Portuguese acronym) and of the Instituto de Medicina Integral Professor Feranando Figueira (IMIP, in the Portuguese acronym), presenting with complaints of oligomenorrhea, when the menstrual cycle occurs at an interval ≥ 35 days, or secondary amenorrhea, when there has been an absence of menstruation over three consecutive cycles or for 6 months, and/or signs of hyperandrogenism, considered as hirsutism. Of these, six were excluded because they presented other endocrinopathies: late-onset congenital adrenal hyperplasia (n = 1), hyperthyroidism (n = 2), hypothyroidism (n = 1), hyperprolactinemia (n = 2), and one because she was breastfeeding. Of these, 111 women aged between 18 and 39 years old were diagnosed with PCOS according to the Rotterdam Consensus, and agreed to participate in the present research.^2^ Forty-five participants were lost because they did not perform all the laboratory tests and/or a pelvic ultrasound scan.

In the anamnesis, the characteristics of the menstrual cycle were investigated, along with age, use of medications and of contraceptive methods. Physical examinations were performed at the first consultation by a single researcher, and the same instruments/equipment (same manufacturers and models) were used at both centers. The height in centimeters (cm) and weight in kilograms (kg) of all the patients were measured without shoes, in orthostatic position, with an anthropometric mechanical scale Filizola (Filizola, Parque Grajaú, SP, Brazil). During the physical examination, the Waist Circumference (WC) was measured using a tape measure midway between the iliac crest and the lower costal border, and blood pressure was measured according to the recommendations of the Seventh Brazilian Guidelines for Hypertension.^25^ An evaluation of hirsutism was performed by the same researcher based on the presence and distribution of terminal hair, according to the modified Ferriman-Gallwey scale (hirsutism was present with a score ≥8).^26^ The women were evaluated after a pause in hair removal of at least 4 weeks, and none of the patients had undergone permanent hair removal procedures.

In the clinical laboratory at each center, blood was collected from patients by venipuncture after fasting for at least 8 hours. This sample was divided into 2 dry test tubes, centrifuged at 3,500 revolutions per minute. In order to analyze the serum hormone levels, one of the test tubes underwent a process of chemiluminescence using an Abbott Architect i2000 (Abbot Laboratories, Chicago, IL, USA); and to evaluate levels of glycemia, triglycerides and high density lipoprotein (HDL) cholesterol, the other test tube was processed by spectrophotometry on the Beckman Coulter Au680 analyzer (Beckman Coulter, Brea, CA, USA). Blood levels were evaluated for glucose, triglycerides, HDL cholesterol, prolactin, 17-hydroxyprogesterone, follicle stimulating hormone (FSH), luteinizing hormone (LH), thyroid stimulating hormone (TSH), free thyroxine (T4), and β-hCG. The oral glucose tolerance test(OGTT) was performed, which evaluates glycemia 2 hours after ingestion of 75 g of glucose. The diagnosis of Impaired Glucose Tolerance was considered if the test value was greater than or equal to 140 mg / dL and less than 200 mg / dL.^27^ Insulin resistance was also evaluated with homeostatic model assessment of insulin resistance (HOMA-IR), and IR was considered as being present with a HOMA-IR ≥ 2.7.^28^

All of the patients underwent ultrasound scans during any stage of the menstrual cycle; non-virgins underwent transvaginal scans, and virgins underwent abdominal scans. The imaging examinations were performed by the same professional in each service, who measured the ovarian volume, and if the value was > 10 cm^3^, classified it as polycystic ovary.^2^

The selected patients were divided into four groups, according to the phenotypes: A: oligo-ovulation or anovulation + hyperandrogenism + polycystic ovaries; B: oligo-ovulation or anovulation + hyperandrogenism; C: hyperandrogenism + polycystic ovaries; D: oligo-ovulation or anovulation + polycystic ovaries.

The main objective of the present study was to evaluate the prevalence of MetS and its components in the different phenotypes of PCOS. Metabolic syndrome was defined according to the consensus held in 2009 by several scientific entities related to the study of cardiovascular diseases and diabetes, which considered the diagnosis of MetS to be the presence of at least three of the following criteria: abdominal obesity (a waist ≥ 80 cm in women), hypertension (systolic blood pressure ≥130 mmHg and/or diastolic ≥ 85 mmHg), high levels of blood glucose (fasting level ≥ 100 mg/dL or a diagnosis of type 2 diabetes), high triglyceride levels (≥ 150 mg/dL or in treatment), and a reduction in HDL cholesterol (< 50 mg/dL or in treatment).^29^

Exclusion criteria were pregnant or lactating women, the use of hormonal contraceptives or of any medications that could have interfered in the hormonal profile over the previous 3 months, as well as the presence of other endocrinopathies associated with anovulation.

The calculation for the sample size proportions, a finite population equal to infinite, was carried out based on the prevalence of a PCOS of 8.5%.^30^ According to these criteria, the sample size for the study was 111 women.

Data were entered into Excel 2010 (Microsoft Corporation, Redmond, WA, USA) and analyzed in the R 3.3.1 statistical software (R Foundation, Vienna, Austria), which is freely available at http://www.r-project.org. The graphs presented here were produced both in R and in Excel. Initially, in the statistical analysis, a descriptive analysis of the study variables was performed. For the continuous variables, we used the mean and median values as measures of central tendency, and the standard deviation (SD) as a measure of dispersion. Initially, in the univariate analyzes, the normality assumptions of the quantitative variables were evaluated with the Kolmogorov-Smirnov test. If the normality assumption was valid, the assumption of homogeneity was evaluated by the Bartlett test, and in the absence of normality, the modified Levene test was applied. A comparison of the variables among the four phenotypes of PCOS was performed using the analysis of variance (ANOVA) test, when the normality and homogeneity assumptions were accepted, and, in the absence of normality, by the Kruskal-Wallis non-parametric test. The Fisher exact test was used for the qualitative variables. The odds ratios (ORs) between the phenotypes for the development of MetS and of IR were estimated with the multivariate analyzes. In order to identify the possible factors associated with MetS and IR, we tested the relationship between these outcomes and the study variables. The association was evaluated through the logistic regression model under the stepwise regression forward selection process. The variables were maintained in the final model when they presented a *p*-value < 0.05, according to the maximum likelihood ratio test. Finally, the prevalence ratios for each of these variables were estimated with their respective confidence intervals (CI), which were of 95%.

All of the patients were informed of the risks and benefits related to the procedures and the research, and together with the researcher, they read the Informed Consent Form (ICF). The research was approved by the Research Ethics Committee at IMIP.

## Results

The clinical, hormonal and metabolic characteristics of the patients with PCOS are presented in Table 1. The prevalence of each phenotype of PCOS presented the following distribution: 54.1% met the criteria of phenotype A; 11.7% of phenotype B; 14.4% of phenotype C; and 19.8% of phenotype D.

**Table 1:** The clinical, hormonal and metabolic characteristics of patients with polycystic ovary syndrome.

Variables	*n*	%
Age (years old)		
18–20	16	14.4
20–25	23	20.7
25–30	37	33.3
30–35	25	22.5
35–40	10	9.1
BMI		
< 25 kg/m^2^	36	32.4
25–30 kg/m^2^	26	23.4
≥ 30 kg/m^2^	49	44.1
Oligomenorrhea/amenorrhea	93	83.8
Hirsutism	87	78.4
AC ≥ 80 cm	73	65.8
HDL < 50 mg/dL	60	54.1
Triglycerides ≥ 150 mg/dL	39	35.1
BP ≥ 130/85 mmHg	25	22.5
Fasting glucose ≥ 100 mg/dL	8	7.2
IR	44	39.6
Impaired glucose tolerance	8	7.2
Total testosterone ≥ 80 mg/dL	11	9.9
PCO	98	83.3
Metabolic Syndrome	34	33.6

Abbreviations: AC, abdominal circumference; BMI, body mass index; BP, blood pressure; HDL, high density lipoprotein; IR, insulin resistance; PCO, polycystic ovary.

Table 2 presents the mean values of the continuous variables in relation to the phenotypes of PCOS, in which it was identified that the mean values of fasting and 2 hours after ingesting glucose (GTT) were higher in phenotype D, with values of 93.6 mg/dL and of 120.0 mg/dL, respectively. The mean value of HDL cholesterol (44.7 mg/dL) was lower in phenotype D, whereas the mean triglycerides level (158 mg/dL) was higher in phenotype A. All of the four variables presented statistical significance (*p* < 0.05).

**Table 2:** The mean (and standard deviation) of the continuous variables being studied

	PCOS Phenotypes				
Variables	A *n* = 60	B *n* = 13	C *n* = 16	D *n* = 22	*p*-value*
FG (mg/dL)	86.5 (8.8)	89.5 (10.8)	80.9 (9.1)	93.6 (16.4)	0.0207
HDL (mg/dL)	51.5 (14.2)	49.2 (9.4)	57.8 (12.3)	44.7 (11.7)	0.0251
Triglycerides (mg/dL)	158 (122.8)	98.4 (38.2)	132.2 (44.9)	117.5 (63.1)	0.0213
AC (cm)	85.2 (16.7)	82.5 (15.8)	83.9 (8.5)	90.11 (15.6)	0.5327
SBP (mmHg)	117 (16.8)	106.9 (18.4)	116.2 (10.2)	114.1 (13.0)	0.2944
DBP (mmHd)	74.8 (11.1)	70.8 (8.6)	76.2 (6.2)	74.3 (10.9)	0.4525
HOMA-IR	3.2 (2.3)	2.7 (2.5)	2.4 (1.5)	3.2 (2.2)	0.4157
OGTT (mg/dL)	112.7 (19.6)	107.9 (19)	102.6 (5.7)	120.0 (31.1)	0.0448
BMI (kg/m^2^)	29.34 (6.51)	27.08 (7.46)	29.15 (4.23)	29.10 (6.91)	0.5385

* Statistically significant comparisons (*p *< 0.05); *Non-parametric Kruskal-Walls Test.

Abbreviations: AC, abdominal circumference; BMI, body mass index; DBP, diastolic blood pressure; FG, fasting glucose; HDL, high-density lipoprotein; HOMA-IR, homeostatic model assessment of insulin resistance; OGTT, oral glucose tolerance test; PCOS, polycystic ovary syndrome; SBP, systolic blood pressure..

Table 3 presents the prevalence of metabolic changes among the phenotypes, and it may be observed that levels of HDL cholesterol < 50 mg/dL are more frequent in phenotype D, and were present in 77.3% of the women in this group. This was the only variable with a statistically significant difference (*p* < 0.05) among the phenotypes.

**Table 3:** The number (and percentages) of metabolic syndrome, of each component of metabolic syndrome, of insulin resistance and of impaired glucose tolerance within each polycystic ovary syndrome phenotype

	PCOS Phenotypes				
Variables	A *n* = 60	B *n* =13	C *n* =16	D *n* =22	*p*-value*
Metabolic syndrome	20 (33.3)	4 (30.8)	2 (12.5)	8 (36.4)	0.3811
FG ≥ 100 mg/dL	3 (5.0)	1 (7.7)	0.0 (0.0)	4 (18.2)	0.1225
HDL < 50 mg/dL	32 (53.3)	7 (53.8)	4 (25.0)	17 (77.3)	0.0155
Triglycerides ≥ 150 mg/dL	25 (41.7)	2 (15.4)	6 (37.5)	6 (27.3)	0.2826
AC ≥ 80 cm	38 (63.3)	7 (53.8)	13 (81.2)	15 (68.2)	0.4457
BP ≥130/85 mmHg	14 (23.3)	3 (23.1)	3 (18.7)	5 (22.7)	0.9844
IR	26 (43.3)	4 (30.8)	3 (18.7)	11 (50.0)	0.1943
Impaired GL tolerance	4 (6.7)	0.0 (0.0)	0.0 (0.0)	4 (18.2)	0.1541
Obesity	32 (53.3)	4 (30.8)	77 (43.7)	6 (27.3)	0.1403

*Statistically significant comparisons (*p* < 0.05); *The Fisher exact test.

Abbreviations: AC, abdominal circumference; BP, blood pressure; FG, fasting glucose; GL, glucose; HDL, high density lipoprotein; IR, insulin resistance; MetS, metabolic syndrome.

In the logistic regression analysis, the variables HOMA-IR, age, and body mass index (BMI) demonstrated an impact on the chance of developing MetS. The OR revealed that obesity increased the chance of developing MetS by approximately 4.8 times (Table 4).

**Table 4:** The odds ratios and 95% confidence intervals for predicting metabolic syndrome based on the logistic regression analyzes

Variables	Positive categories	OR (95%CI)	p-value*	AIC*
HOMA-IR	−	1.435 (1.083–1.904)	0.0120	
Age	−	1.159 (1.049–1.281)	0.0037	103.81
BMI	−	1.114 (1.011–1.227)	0.0290	
IR	HOMA-IR ≥ 2.7	2.595 (1.043–6.459)	0.0403	121.06
Obesity	BMI ≥ 30	4.851 (1.907–12.340)	0.0009	

Abbreviations: AIC, Akaike information criterion; BMI, body mass index; CI, confidence interval; HOMA-IR, homeostatic model assessment of insulin resistance; IR, insulin resistance; MetS, metabolic syndrome; OR, odds ratio.

In the logistic regression analysis, to evaluate the impact that each phenotype exerted over the chance of developing MetS, no statistically significant association was observed. However, when evaluating the impact that each phenotype exerted on the risk of developing MetS in the group of women with an AC ≥ 80 cm, the risk of developing MetS in phenotype A increased approximately six-fold in relation to phenotype C (Table 5).

**Table 5:** The risk ratio of developing metabolic syndrome amongst the polycystic ovary syndrome phenotypes in the group of women with an abdominal circumference ≥ 80 cm

	Phenotype *x* in relation to phenotype *y*			
*x*	*y*	OR	95% CI	*p*-value*
A	B	0.833	(0.164–4.239)	0.8261
A	C	6.111	(1.191–31.366)	0.0301
A	D	0.972	(0.294–3.220)	0.9632
B	A	1.200	(0.236–6.105)	0.8261
B	C	7.333	(0.877–61.327)	0.0660
B	D	1.167	(0.191–7.116	0.8673
C	A	0.164	(0.032–0.840)	0.0301
C	B	0.136	(0.016–1.140)	0.0660
C	D	0.159	(0.026–0.978)	0.0473
D	A	1.029	(0.311–3.407)	0.9632
D	B	0.857	(0.141–5.228)	0.8673
D	C	6.286	(1.022–38.648)	0.0473

Abbreviations: CI, confidence interval; OR, odds ratio.

## Discussion

In the present study, it was identified that the classic phenotypes, composed of A and B, were the most frequent, followed by the non-hyperandrogenic (D), and then by the least frequent, the ovulatory (C), which is compatible with results observed in other studies.^3^ ^10^ ^16^ However, the prevalence of phenotype D varies considerably among studies. In a study conducted by Ladrón de Guevara et al,^20^ who evaluated 220 Chilean women and 206 Argentinian women with PCOS, phenotype D (non-hyperandrogenic) was the least prevalent, and corresponded to 1% and to 10% in each country, respectively. Clark et al^31^ encountered 11% of the participants with the D phenotype, while Diamanti-Kandarakis et al^32^ discovered phenotype D in 6.78% of the participants.

This variation in the prevalence of phenotype D (non-hyperandrogenic) among studies may be due to the subjectivity involved in evaluating hirsutism, a relevant sign for evaluating clinical hyperandrogensim. The Ferriman and Gallwey scale presents low reproducibility with great interobserver variability, which may reach 50%, depending on the area being considered.^33^ However, it is a widely used instrument in the clinical practice because it is easy to use and the costs involved are low.^34^

The prevalence of MetS encountered in the present sample was 33.6%, with no statistically significant difference between the phenotypes. However, a lower prevalence was observed in phenotype C (ovulatory). In the group of women with an AC ≥ 80 cm, we observed that in phenotype A, the risk of developing MetS increased approximately six-fold in relation to phenotype C (ovulatory), which is also corroborated in the literature.^3^ ^4^ ^20^

In the present study, the prevalence of IR in women with PCOS was 39.6%, which is a comparable rate with that reported in the literature, which ranges from 25 to 70%.^9^ ^16^ ^17^ However, when comparing the phenotypes, no statistically significant difference was observed in the prevalence of IR among the four groups. This finding differs from other studies, which report a higher frequency of IR in the classic phenotypes (A and B), attributing a relevant role to the excess of androgen in the development of central obesity and in an exacerbation of IR.^3^ ^13^ ^16^ ^20^ ^21^ ^22^ ^32^ ^35^

By evaluating each metabolic change separately, it may be observed that triglycerides ≥ 150 mg/dL were more prevalent in phenotype A. We also identified that fasting glycemia ≥100 mg/dL, decreased glucose tolerance, HDL cholesterol < 50 mg/dL, and IR were also found to be more frequent in phenotype D. With the exception of HDL cholesterol, the other variables did not present statistical significance (*p *< 0.05). It should be noted that the D phenotype group presented with a higher prevalence of an increased AC, of which 68.2% demonstrated an AC ≥ 80 cm and presented the highest mean AC (90.1 cm), which may justify the higher prevalence of metabolic changes encountered within this group. This finding indicates the preponderant role of abdominal obesity in developing metabolic changes. The interrelations between PCOS and obesity are complex. However, two important aspects may be highlighted: 1–hyperandrogenism, which increases the expression of genes involved in lipogenesis, with a predisposition for fat accumulation, particularly in the abdominal cavity; 2 - IR with compensatory hyperinsulinemia, which stimulates androgen production in the ovaries and in the adrenal glands, thereby closing the feedback loop.^17^ ^19^ ^36^ A laboratory evaluation for hyperandrogenism was performed by determining the total blood levels of testosterone. Studies consider the measurement of free testosterone or free testosterone index as the most sensitive measures to assess hyperandrogenemia.^2^ ^34^ To evaluate the ultrasound scan of polycystic ovary, we only considered an ovarian volume > 10 cm^3^. It was not possible to obtain the follicular counting information, as recommended by the Rotterdam Consensus.^2^ These characteristics may represent methodological limitations of the present study.

## Conclusion

The classic phenotypes of PCOS, composed of A and B, were the most frequent, followed by the non-hyperandrogenic (D) and the ovulatory (C). The prevalence of MetS and IR among the PCOS phenotypes did not present statistically significant differences. Abdominal obesity played a significant role in the development of metabolic changes, irrespective of the PCOS phenotype. Prospective studies are needed to identify which clinical, hormonal and metabolic characteristics of each phenotype in PCOS may be considered predictive factors for the onset of MetS.
